# PEComa with metastatic pulmonary embolism upon presentation: A case report

**DOI:** 10.1016/j.radcr.2025.06.009

**Published:** 2025-06-24

**Authors:** Thomas Saliba, David Rotzinger, Naïk Vietti-Violi, Emanuele Avola

**Affiliations:** Centre Hospitalier Universitaire Vaudois (CHUV), Lausanne, Switzerland

**Keywords:** PECOMA, Metastasis, Lung, Vascular, Embolism

## Abstract

Background: Perivascular epithelioid cell tumors (PEComas) are rare mesenchymal neoplasms with an incidence of approximately 0.3 per million. They exhibit histological and immunohistochemical features that overlap with angiomyolipomas and clear cell tumors. PEComas predominantly affect women and are either classified as being linked to tuberous sclerosis or are classified as PEComas-Not Otherwise Specified which means that their origin is not linked to tuberous sclerosis. They often arise from the genitourinary tract. The malignant potential of these tumors is determined by factors such as size (>5 cm), mitotic activity, necrosis, and vascular invasion. Treatment involves resection and mTOR inhibitors. Standardized treatment protocols are lacking due to rarity of the pathology, so each occurrence is considered on a case-by-case basis. A 69-year-old woman with dyspnea underwent CT imaging, revealing a large renal mass, a liver lesion, lung nodules, enlarged hilar lymph nodes, and a pulmonary embolism. Subsequent FDG-PET-CT confirmed FDG-avid lesions, with uptake of the pulmonary emboli suggesting their malignant origin. A subsequent liver biopsy diagnosed a malignant PEComa. MRI demonstrated T2 hyperintensity, restricted diffusion and postcontrast enhancement. Treatment with Everolimus resulted in a partial response of all lesions on 3-month follow-up imaging. PEComas were first recognized by the World Health Organization in 2002 and often present with nonspecific symptoms. They may manifest with metastatic disease at the time of diagnosis. This case highlights metastatic pulmonary embolism as an initial presentation. PEComas pose diagnostic challenges due to their nonspecific imaging findings and varied presentations. This case underscores their potential for aggressive behavior and the role of mTOR inhibitors. Despite successful treatment, the prognosis remains variable, necessitating multidisciplinary management and long-term surveillance. Early biopsy and molecular profiling are critical for optimizing outcomes. With this case report, we hope to bring attention to the possibility of pulmonary emboli being of metastatic origin when found in the context of a pre-existent oncological disease.

## Introduction

Perivascular epithelioid cell tumours (PEComas) are a family of rare mesenchymal tumours, composed of perivascular epithelioid cells, with an incidence of around 0.3 per million [[1], [2], [3]]. These tumors share histological similarities with clear cell ”sugar” tumours of the lung and angiomyolipomas (AML) [1,2,4]. The incidence of PEComas is 7 times greater in women, with a peak between the second and fourth decades of life [1,2].

Bonetti et al proposed a connection between PEComas and tuberous sclerosis, laying the basis for their classification [2,4]. PEComas are typically classified into 2 groups: those associated with tuberous sclerosis, the most common type, and those not linked with tuberous sclerosis, referred to as “PEComas not otherwise specified” (PEComas-NOS) [1]. Among PEComas-NOS, the majority originate from the genitourinary tract, with 40% coming from gynecological sites [1].

Histologically, PEComas are composed of perivascular epithelioid cells with small, ovoid/round nuclei and clear or a granular, eosinophilic, cytoplasm [1,2]. These cells are located in perivascular spaces with cells arranged into nests, sheets or trabeculae and express melanocytic and smooth muscle cell markers [1,2,5].

As PECOmas are of mesenchymal origin, they can be found anywhere in the body, making them difficult to diagnose based on location [1]. A biopsy is therefore required to establish the diagnosis due to their nonspecific imaging characteristics and clinical presentation.

As these tumors carry the potential for malignancy, resection is the preferred treatment alongside mTOR (mammalian target of rapamycin) inhibitors, though there is no standard of care [1].

## Case presentation

A 69-year-old woman presented to the emergency department. The patient complained of hemoptysis, which had been increasing in frequency over the previous 10 days, with 3 instances of hemoptysis of fresh blood in the last 24 hours. The patient also reported worsening dyspnea. Her clinical examination did not reveal any anomalies. The patient had normal blood pressure, a normal heart rate, a normal temperature (37.2°C) and an oxygen saturation of 96% in ambient air.

The patient denied any previous episodes of dyspnea or thoracic pain. However, she admitted smoking infrequently (2 pack years).

Her personal history included a hysterectomy the previous year for a cervical lesion, but she was unable to provide any further information.

When questioned regarding her family history, she recalled that her mother had died of lung cancer.

A blood test revealed mildly elevated CRP at 24 mg/l (normal: < 5 mg/l) and slight anemia of chronic disease with a hemoglobin level at 111g/l (normal: 115-165 g/L), Ferritin of 332 µgram/l (normal: 15-150 µgram/l) and decreased Iron at 5.2 µmol/l (normal: 10.7-21.4 µmol/l) but normal Folate (39.5 ng/mol, normal: 8.8-60.8 ng/mol) and Vitamin B12 levels (382 pmol/l, normal:145-569 pmol/l).

The prothrombin time was 107 (normal: 75%-125%) and INR was 1.

After a thorough examination, the patient’s hemoptysis, which was her most pressing symptom, was treated with inhaled tranexamic acid.

**Fig. 1:**
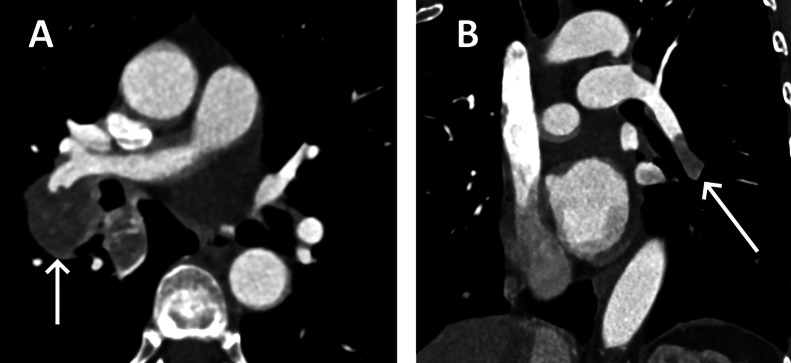
Contrast-enhanced CT of the lungs showing enlarged hilar lymph nodes (A, arrow) and embolism in a segmental pulmonary artery that was later shown to be tumoral in nature (B, arrow).

**Fig. 2:**
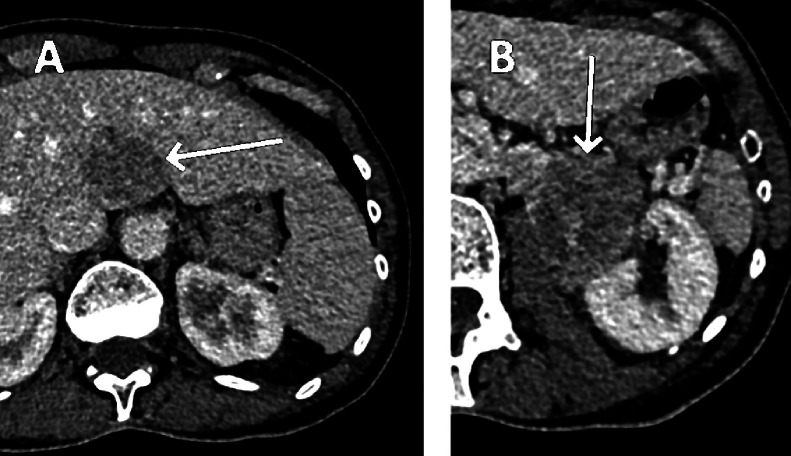
Contrast-enhanced CT of abdomen, acquired during the portal venous phase, showing a liver lesion, later revealed to be a metastasis (A, arrow) and the primary para-renal PEComa (B, arrow).

A contrast-enhanced CT scan was then performed to search for the cause of the dyspnea. This revealed a large enhancing left renal mass, representing the primary tumor (Fig. 1, Fig. 2). Furthermore, a liver lesion, segmental pulmonary emboli, multiple enlarged hilar lymph nodes and multiple angiocentric lung nodules were found (Fig. 1, Fig. 2).

The discovery of a hepatic mass prompted a viral panel for hepatitis A,B,C,E and HIV, which were all negative.

As the patient was unable to receive anticoagulant treatment due to hemoptysis, a doppler ultrasound of the venous system was performed to search for a thromboembolic cause, but none was found.

**Fig. 3:**
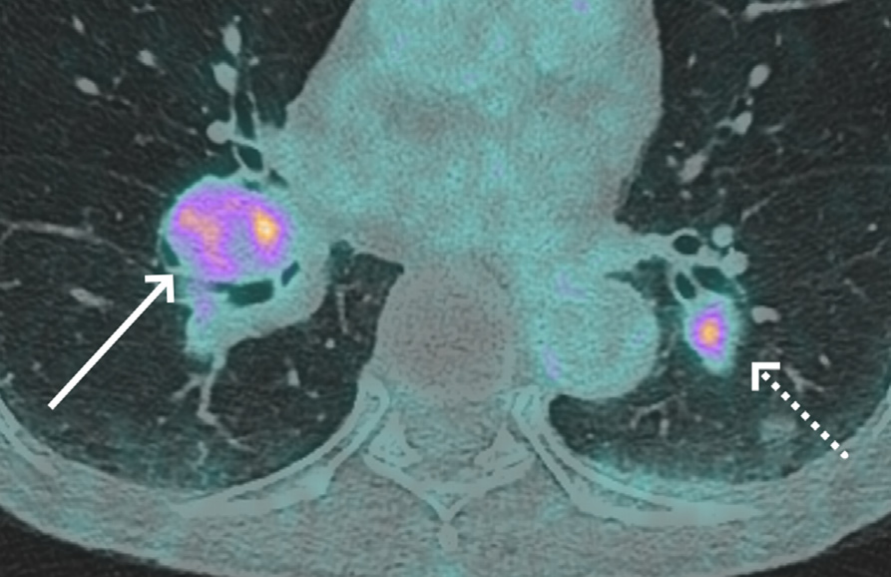
PET-CT showing hyperactivity in the hilar lymph nodes (arrow) and within the segmental embolism (dotted arrow), proving its tumoral nature.

Due to the suspicion of pulmonary mass with multiple metastases, the patient was referred to our tertiary reference center for further investigation. An FDG-PET-CT was performed 2 days later, demonstrating FDG uptake of all of the abdominal lesions, confirming their tumoral nature. Furthermore, there was also uptake of the pulmonary embolism, confirming its metastatic nature (Fig. 3).

To further the diagnostic procedure, a percutaneous liver biopsy was performed. Histopathological analysis of the biopsy revealed epithelial cells with atypical and deformed nuclei, alongside eosinophilic cytoplasm. There were numerous cells undergoing mitosis, with some anomalies being noted. Immunohistochemistry revealed the expression of smooth muscle actin, Melan-A and focal expression of pancytokeratin AE1/AE3. Ki-67 tests revealed increased cellular proliferation. This was compatible with a malignant PEComa with characteristics of an epithelioid angiomyolipoma.

**Fig. 4:**
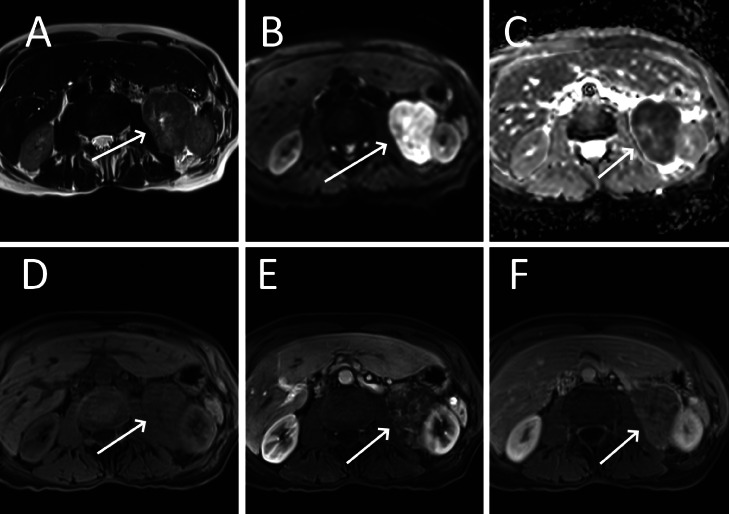
MRI of a para-renal tumor (arrows) that is heterogeneously hyperintense in T2-weighted imaging (A), restricts diffusion on high b-value (B), is hypointense in ADC imaging (C), is isointense in T1-weighted imaging (D) but enhances arterial (E) and venous phases (F).

**Fig. 5:**
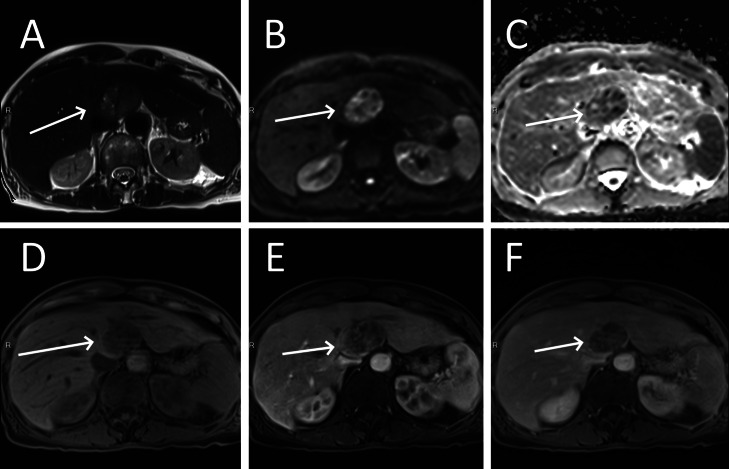
MRI of a hepatic metastasis (arrows) that is heterogeneously hyperintense in T2-weighted imaging (A), restricts diffusion on high b-value(B), is hypointense in ADC imaging (C), is hypointense to the liver in T1-weighted imaging (D) and enhances heterogeneously in arterial (E) and venous phases (F).

An abdominal MRI performed to further characterize the renal mass, showing that both the renal and liver masses were hypointense on T1 weighted imaging (WI) and demonstrated increased venous phase enhancement relative to adjacent parenchyma after gadolinium injection. Both lesions exhibited restricted diffusion and were heterogeneously hyperintense on T2 WI (Fig. 4, Fig. 5). Based on this, and histology of the liver lesion, the diagnosis of a renal PEComa.

The patient was treated with Everolimus at 10 mg/day and discharged pending follow-up exams.

**Fig. 6:**
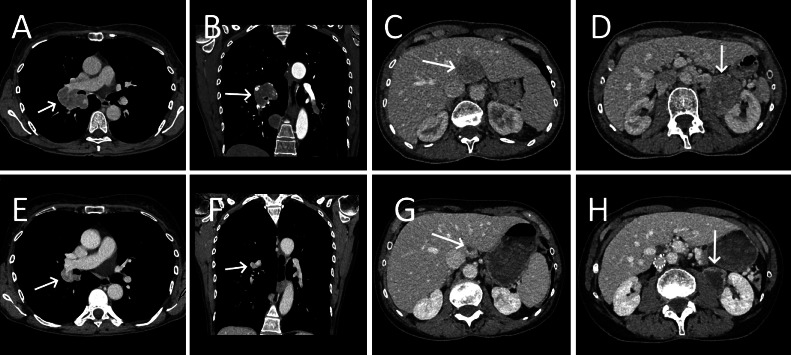
Response to the therapy of the hilar metastasis (Before treatment A and B, arrows; Post treatment E and F, arrows), the liver metastasis (Before treatment C, arrow; Post treatment G, arrow) and the para-renal tumor (Before treatment D, arrow; Post treatment H, arrow).

A follow-up CT scan 3 months later showed an excellent response to the treatment with all the lesions regressing in size and some disappearing completely (Fig. 6), including pulmonary embolism, despite the lack of anticoagulation therapy, confirming its tumoral origin.

The patient continues to undergo monthly clinical and biological follow-up, and tri-monthly CT-scans.

## Discussion

Perivascular epithelioid cells were first discovered in renal AMLs by Apitz in 1943, with the term PEComa being first introduced by Zamboni in 1996 whilst describing a pancreatic tumor [2]. These tumors were officially recognized by the World Health Organization in 2002 as a family of neoplasms characterized by the immunohistochemical and morphological features of perivascular epithelioid cells [2]. Due to their histopathological similarities with other tumors, immunohistochemistry is essential for establishing a definitive diagnosis [6].

PEComas typically present with nonspecific symptoms, such as focal pain [2]. However, they may also cause symptoms specific to their site of origin, such as vaginal bleeding or hemoperitoneum in case of uterine PEComas or hemoptysis for pulmonary tumors [1,7]

PEComas are often discovered incidentally, being found during imaging performed in the search for unrelated pathologies [2]. Our literature search identified only 1 other where a cardiac PEComa was discovered during a CT exam performed for suspected pulmonary embolism. In that case, a cardiac PEComa was found that extended into the left pulmonary vein, occluding it [8]. However, we believe our case to be unique, as the patient initially presented with metastatic emboli occluding a pulmonary artery, though there were no signs of vascular invasion.

While most PEComas are benign, some can become malignant. Key markers of malignancy include size > 5 cm, increased mitotic rates, high vascularity, vascular invasion, infiltrative growth patterns and necrosis [2,9]. Tumors lacking these characteristics are considered benign, whereas those with 2 or more malignant features are classified as having a malignant disease, as was the case of our patient [2]. Notably, more recent classifications have simplified the criteria for malignancy to include only size > 5 cm and increased mitotic rates, as these were the only significant predictors of recurrence following resection [5]. PEComas most commonly metastasize to the lungs, followed by the liver and peritoneum, though they can be found in nearly any tissue [2,10].

PEComas are typically hypodense or isodense on CT, and enhance in the venous phase after contrast injection, except in cases of necrosis [10]. The enhancement can be either heterogenous or homogenous, sometimes revealing vascular invasion [10]. On MRI, PEComas appear iso- or hypointense on T1-weighted imaging (T1WI), but exhibit will enhance in the venous phase after gadolinium administration in a similar manner to a CT exam [10,11]. The masses will be heterogeneously hyperintense on T2WI [10,11]. Notably, some tumors may include spontaneously T1WI hyperintense foci, which can be attributed to macroscopic fat or hemorrhage [10,11]. These tumors typically display restricted diffusion [1]. Our patient presented with imaging characteristics typical of PEComas, with the notable absence of hemorrhage.

Due to the rarity of PEComas, there are no established treatment guidelines.

The mainstay of PEComa treatment in nonmetastatic disease is complete surgical resection [12]. When complete resection is not achievable, chemotherapy, mTOR (mammalian target of rapamycin) inhibitors and VEGFR (Vascular Endothelial Growth Factor) inhibitors can be used [12]. Everolimus, the treatment used for our patient, has demonstrated effectiveness in some cases, with a clinical response observed in 4 out of 5 patients included in a trial [12]. Patients that are not responsive to the first line of treatment should benefit from a multidisciplinary discussion to determine the next steps [12]. Radiotherapy is generally reserved for symptom relief in palliative therapy [12].

Treatment outcomes for PEComa patients are variable but generally favorable [13]. One study of 189 patients found that 21,1% had evidence of recurrence, with only 10,6% dying from the PEComa. However, this data included many patients with cutaneous PEComas, which typically have better outcomes [5]. Other studies have shown that metastatic disease develops in approximately 20-50% of cases [13]. This shows that there is high heterogeneity with regard to outcome, based on the study design and affected organ.

## Conclusion

We presented the first case of a PEComa presenting with a metastatic pulmonary embolism. PEComas are rare tumors that can arise from nearly any tissue but have a predisposition for the genitourinary tract. They can be clinically silent and may only be discovered incidentally during imaging performed in the context of unrelated pathologies. When identified, the primary treatment is surgical excision, though chemotherapy and immunotherapy have been successfully used in patients with metastatic disease. Despite treatment, the prognosis remains variable, with up to half of patients developing distant metastatic disease despite treatment.

## Patient consent

We hereby declare that we the patient provided informed consent that their data could be used for scientific purposes.
